# Circular RNA hsa_circ_0076248 promotes oncogenesis of glioma by sponging miR‐181a to modulate SIRT1 expression

**DOI:** 10.1002/jcb.27966

**Published:** 2018-12-03

**Authors:** Bingxi Lei, Yutao Huang, Zhiwei Zhou, Yiying Zhao, Ashish Jung Thapa, Wenpeng Li, Wangqing Cai, Yuefei Deng

**Affiliations:** ^1^ Department of Neurosurgery Sun Yat‐sen Memorial Hospital, Sun Yat‐sen University Guangzhou China

**Keywords:** glioma, hsa_circ_0076248, microRNA‐181a, oncogenesis, silent information regulator 1

## Abstract

Glioma is one of the most common primary malignancies of the central nervous system, which has aggressive clinical behavior and a poorer prognosis. MicroRNAs (miRs) are a class of small noncoding RNAs that function as mediators of gene expression, which can be sponged by circRNA provided with a closed circular structure. Dysregulations of circular RNAs (circRNAs) and miRs have been implicated in the development and progression of glioma. In the current study, we investigated the role of circular RNA hsa_circ_0076248 in mediating the oncogenesis of glioma by sponging miR‐181a to modulate silent information regulator 1 (SIRT1) expression in vitro and in vivo. The quantitative real‐time polymerase chain reaction results showed that the expression of miR‐181a was significantly decreased in glioma tissues and cell lines compared with normal brain tissues and normal gliocyte, respectively, and the expression of hsa_circ_0076248 and SIRT1 demonstrated the opposite. Bioinformatics analysis identified hsa_circ_0076248 could sponge miR‐181a, and miR‐181a could target the mRNA of SIRT1. Our results verified that downregulating hsa_circ_0076248 or upregulating miR‐181a could depress the proliferation and invasion of glioma in vitro and in vivo. The experiment also showed that downregulating hsa_circ_0076248 or upregulating miR‐181a could remarkably promote the temozolomide chemotherapy sensitivity. Furthermore, Western blot analysis testified that downregulating hsa_circ_0076248 or upregulating miR‐181a could promote the expression of p53 and SIRT1. In summary, our study sheds light on the regulatory mechanism of hsa_circ_0076248 in glioma growth and invasion via sponging miR‐181a, which downregulates the SIRT1 expression and also suggests that hsa_circ_0076248, miR‐181a, and SIRT1 may serve as potential therapeutic targets for glioma.

## 1 INTRODUCTION

Glioblastoma multiforme (GBM) is the most frequent primary tumor in the central nervous system, and patients with malignant glioma have a very poor prognosis.^1^, ^2^ Because of the chemotherapy resistance, there is no mature or effective method for GBM therapy.^3^, ^4^ Currently, GBM is still being treated by means of radical surgery followed by radiotherapy and chemotherapy.^5^, ^6^, ^7^ It appears that conventional drugs, such as temozolomide, have therapeutic benefits in prolonging the survival of GBM patients.^8^, ^9^, ^10^ Targeted therapies against a genetic anomaly using specific small molecule inhibitors are now in clinical trials.^11^, ^12^, ^13^ Multiple genetic changes in GBM suggest that a single drug is unlikely to offer an effective and complete solution to the problem; new GBM therapy target searching a new focus recently, has gradually been in the hot research these years.

Circular RNAs (circRNAs) are a new class of noncoding RNAs with a closed circular structure, which can sponge up to 120 microRNAs (miRNAs) as a competitive endogenous RNA (ceRNA).^14^ MicroRNA (miRNA) is a type of endogenous RNA about 20 to 24 nucleotides in length. Recent studies have disclosed various critical roles of circRNAs and miRNAs in cell growth and apoptosis.^15^ Each miRNA may have multiple target genes and several miRNAs often target the 3′‐untranslated regions (3′‐UTR) of the genes and subsequently decrease their expression. Recent studies indicate that circRNAs are more enriched in neuronal tissues and their expressions are often regulated during neuronal development or on tumorigenesis.^16^, ^17^, ^18^, ^19^, ^20^, ^21^ The miRNAs perform important functions in various oncogenic signaling pathways, such as cell proliferation and migration.^22^ MiR‐181 is a critical modulator for the development of T cell and natural killer cells^23^, ^24^ and is involved in the initial and progression of many tumors, like nasopharyngeal carcinoma, breast cancer, and hepatic carcinoma.^25^, ^26^, ^27^ MiR‐181 family contains a, b, c, and d isoforms, downregulation of hsa‐miR‐181a has been found to be involved in oncogenesis of many cancers, including glioma.^28^ Human hsa_circ_0076248 also named hsa‐circ‐ZFAND3.7, one of the circular structures of ZFAND3 transcripts, acting as functional RNA has been poorly studied and reported until nowadays. Bioinformatics analysis identified hsa_circ_0076248 could sponge miR‐181a and miR‐181a could target the mRNA of silent information regulator 1 (SIRT1).

SIRT1 was a conserved nicotinamide adenine dinucleotide (NAD)‐dependent protein deacetylase that regulates lifespan.^29^, ^30^, ^31^, ^32^ Recent studies have shown that mammalian SIRT1 was emerging as a key regulator of cell survival in the face of cellular stresses which otherwise triggered apoptotic pathways.^33^, ^34^ SIRT1 was also involved in cell growth, apoptosis, and tumorigenesis.^35^, ^36^, ^37^, ^38^ Recent studies demonstrated overexpression of SIRT1 in cancer tissue, compared with normal tissue, suggesting that SIRT1 may act as a tumor promoter.^39^, ^40^, ^41^, ^42^ However, the regulatory pathway of SIRT1 in human glioma and its role was not very clear. In this study, SIRT1 was a target of miR‐181a; the expression levels and the biological function of SIRT1 in human glioma were regulated by miR‐181a. Our study put forward to the regulatory mechanism among hsa_circ_0076248, miR‐181a, and SIRT1 in glioma growth and invasion and suggests that hsa_circ_0076248, miR‐181a, and SIRT1 may serve as potential therapeutic targets for glioma in the future.

## 2 MATERIALS AND METHODS

### 2.1 Tissue samples

Human glioblastoma samples were obtained from surgery operations of Sun Yat‐sen Memorial Hospital, Sun Yat‐sen University. The normal brain tissues were obtained from the patients of traumatic brain edema that underwent partial brain resection, which was preserved in liquid nitrogen. All procedures related to acquiring patient samples were informed consent obtained for all patients and all procedures were approved by the Ethics Committee of Sun Yat‐sen Memorial Hospital Institutional Review Board.

### 2.2 Cell lines and transfection

As cell culture, U251, U87, and HEB cells (Shanghai Institutes for Biological Sciences of Chinese Academy of Sciences, SIBS, Shanghai, China) were cultured under the conditions as instructed by the manufacturer in Dulbecco modified Eagle medium (DMEM; Gibco, BRL) containing 4.5 g/L glucose, with 10% fetal bovine serum (FBS; Gibco, Grandisland, NY) at 37°C in a humidified atmosphere with 5% CO_2_. The medium was replaced every 3 days. Cells were transiently transfected with the si‐hsa_circ_0076248, miR‐181a‐inhibitor, or miR‐181a‐mimics and their negative‐control (NC) sequence, respectively. The small interfering RNA (siRNA), which silenced the hsa_circ_0076248 was named si‐hsa_circ_0076248 and its negative control named NC (si). The RNAs which can upregulate and downregulate the miR‐181a were defined as miR‐181a‐mimics and miR‐181a‐inhibitor, respectively and their negative control was defined as NC (inhibitor). The si‐hsa_circ_0076248, miR‐181a‐inhibitor, or miR‐181a‐mimics and their negative control are variety RNAs and their sequences used in this study were the following: si‐hsa_circ_0076248, sense, 3′‐dTdTCUCAUUUCUCCUCAUAAAA‐5′, antisense, 5′‐GAGUAAAGAGGAGUAUUUUCA‐3′; miR‐181a‐mimics, 5′‐AACAUUCAACGCUGUCGGUGAGU‐3′, 5′‐UCACCGACAGCGUUGAAUGUUUU‐3′; miR‐181a‐inhibitor, 5′‐ACUCACCGACAGCGUUGAAUGUU‐3′; NC (si), sense, 5′‐UUCUCCGAACGUGUCACGUTT‐3′, antisense, 5′‐ACGUGACACGUUCGGAGAATT‐3′; NC (inhibitor), 5′‐CAGUACUUUUGUGUAGUACAA‐3′ (GenePharma, Shanghai, China).

### 2.3 Reverse‐transcription and real‐time polymerase chain reaction

Total RNAs were isolated from the cells using TRIzol reagent (TaKaRa, Kusatsu, Japan). The RNA was subsequently treated with RNase‐free DNase I (Roche, Germany, Switzerland). Synthesis of the complementary DNA was done by using the BcaBest RNA PCR kit from Takara according to the manufacturer's instructions. Quantitative real time polymerase chain reaction (qRT‐PCR) was carried out using the iQ5 Multicolor Real‐Time PCR Detection System (Bio‐Rad Laboratories, Hercules, California) with Real‐Time PCR Master Mix (TaKaRa). The sequences of primers used in this study were the following: hsa‐circ‐0076248, forward 5′‐CCTCGATAACCACGCCAACT‐3′, reverse 5′‐TGGAGCGGAATCATCGTCTG‐3′; miR‐181a reverse transcript primer (stem‐loop method): 5′‐GTCGTATCCAGTGCGTGTCGTGGAGTCGGCAATTGCACTGGATACGACACTCAC‐3′; forward primer, 5′‐GCAACAUUCAACGCUGUCG‐3′, reverse primer, 5′‐GTGCAGGGTCCGAGGT‐3′; SIRT1, forward, 5′‐ATCTGACTTTGCTCCCCTTAACC‐3′, reverse, 5′‐GGGCCCTGGTTGCAAGA‐3′. Dihydronicotinamide‐adenine dinucleotide phosphate (NAPDH), forward, 5′‐CAATGACCCCTTCATTGACC‐3′, reverse, 5′‐GACAAGCTTCCCGTTCTCAG‐3. Expression of each gene was quantified by measuring *C*_t_ values and normalized using the $2^{-\Delta \Delta C_{t}}$ method. The program was as following: Stage 1, Reps: 1, 95℃, 30 seconds; Stage 2, Reps: 40, 95℃, 5 seconds, 60℃, 30 seconds; Stage 3, Reps: 1, 95℃, 15 seconds, 60℃, 60 seconds.

### 2.4 Western blot analysis assay

The protein concentration was determined using the BCA Protein Assay Kit (Pierce Chemical, Rockford, IL). Protein was separated with sodium dodecyl sulfate polyacrylamide gel electrophoresis, transferred to a polyvinylidene difluoride (PVDF) membrane (Life Technologies, San Diego, CA). The PVDF membrane was incubated with SIRT1 monoclonal antibody (rabbit; 1:2000; Abcam, ab32441) or p53 monoclonal antibody (mouse,1:2000; Abcam). After that, the PVDF membrane was incubated with the mouse antirabbit secondary antibody (1:2000; Abcam) and anti‐mouse secondary antibody (1:2000; Abcam). The immune complexes were detected using the ECL Western blot analysis Kit (Pierce Chemical). All of these assays were independently repeated at least three times.

### 2.5 Cell cycle measured with propidium iodide by flow cytometry

U251 cells were cultured in six‐well plate processed with NC (inhibitor), inhibitor, mimics, NC (si), si, si+inhibitor, according to manufacturer's instruction, respectively. Seventy‐two hours later, cells were gathered and washed with phosphate‐buffered saline (PBS; Gibco, BRL), and then 2 ml of 70% ice alcohol (ethanol:H_2_O = 7:3, −20℃) was added in. Eight hours later, cells were gathered and washed with PBS one time and then 50 µL RNase and 450 µL propidium iodide (PI; Beyotime Biotechnology, Shanghai, China) were added in for analysis of flow cytometer immediately. All of these assays were independently repeated at least three times.

### 2.6 Colony formation assay

U251 cells were cultured in six‐well plate (3‐5 × 10^2^/mL) processed with NC (inhibitor), inhibitor, mimics, NC (si), si, si+inhibitor, according to manufacturer's instruction, respectively. Fifteen days later, cells were fixed with 4% paraformaldehyde and stained with 0.1% crystal violet solution and then the number of colonies on each well was counted. All of these assays were independently repeated at least three times.

### 2.7 Temozolomide toxicity assay

U251 cells were exposed to temozolomide (TMZ) to induce cell death mediated by oxidative damage. 300 μM of temozolomide (TMZ) was added to cell cultures for 72 hours and then cells were incubated in CCK‐8 following the manufacturer's instructions, whose optical density values were subsequently detected by a microplate reader (Multiskan MK3; Thermo Fisher Scientific, San Diego, CA). All assays were performed in octuplicate and repeated at least three times.

### 2.8 Annexin V‐APC‐A/PI apoptosis assay

U251 cells were cultured in a six‐well plate processed with NC (inhibitor), inhibitor, mimics, NC (si), si, si+inhibitor, according to manufacturer's instruction, respectively, accompanied by 300 µM TMZ. Seventy‐two hours later, cells were gathered and washed with PBS, and then a 400‐µL binding buffer of Annexin V‐APC‐A/PI kit (Beyotime Biotechnology) was added in. Then the liquid was divided into two groups one of which contains 200 µL liquid to adjacent flow cytometer and the other of which is added with 5 µL Annexin V‐APC‐A for 15 minutes in the dark and subsequently 10 µL PI to test with flow cytometer immediately: exciting light (630 nm) and reception (600 nm) to test APC‐A and *λ* > 560 nm to test PI. All of these assays were independently repeated at least three times.

### 2.9 Scratch test

U251 cells were cultured in six‐well plate processed with NC (inhibitor), inhibitor, mimics, NC (si), si, si+inhibitor, according to manufacturer's instruction, respectively. Seventy‐two hours later, the culture media was aspired and free‐serum DMEM was added in to hungry cells for 12 hours so that the scratch test can exactly reflect the “right migration” caused by the cell movement rather than the "false migration" caused by the proliferation. A single linear scratch was made through the center of the plate with a 200 µL tips and then photographed at 0, 12, 24, 36, 48, 60, 72 hours under an inverted microscope. All of these assays were independently repeated at least three times.

### 2.10 Transwell invasion test

U251 cells were cultured in six‐well plate processed with NC (inhibitor), inhibitor, mimics, NC (si), si, si+inhibitor, according to manufacturer's instruction, respectively. Seventy‐two hours later, cells were gathered and resuspended with serum‐free DMEM (1 × 10^5^/mL), and then 200 µL were seeded on polycarbonate membrane inserts in a 24‐well transwell apparatus (Costar, Cambridge, MA). In the bottom reservoir, 500 µL of conditioned medium containing 10% FBS was added as a chemoattractant. After the cells were incubated for 24 hours at 37°C in a 5% CO_2_ atmosphere, the inserts were removed from the transwell plates and were washed with PBS. The cells on the top surface of the insert were removed with a cotton‐wool bud, while the cells that were adherent to the underside of the membrane were fixed with 4% paraformldehyde and were stained with 0.1% crystal violet solution. The number of cells on each underside of the membrane was counted under an inverted microscope. All of these assays were independently repeated at least three times.

### 2.11 Animal studies

U251 cells were suspended in serum‐free DMEM (1 × 10^6^ to 1 × 10^7^/mL) and then implanted subcutaneously into four male and four female BALB/c nude mice (200 µL per mice) aged 3 weeks (Beijing Vital River Laboratory Animal Technology, Beijing, China). Two weeks later, the mice were divided into two groups with two male and two female mice randomly, one of which processed with NC (si) and the other si for 5 days continuously. A month later, the BALB/c nude mouse were killed, photographed and dissected to take out the subcutaneous implant tumor whose weight was measured and recorded. Pathological sections were made and observed under a microscope after hematoxylin and eosin staining.

### 2.12 Statistical analysis

Statistical differences were analyzed using the Student *t* test for unpaired samples. An analysis of variance followed by the Dunnett test was used for multiple comparisons with one control group. The criterion for significance (*P* value) was set as mentioned in the figure legends.

## 3 RESULTS

### 3.1 MiR‐181a was downregulated in glioma tissues and cell lines while the circ_0076248 and SIRT1 were upregulated

**Figure 1:**
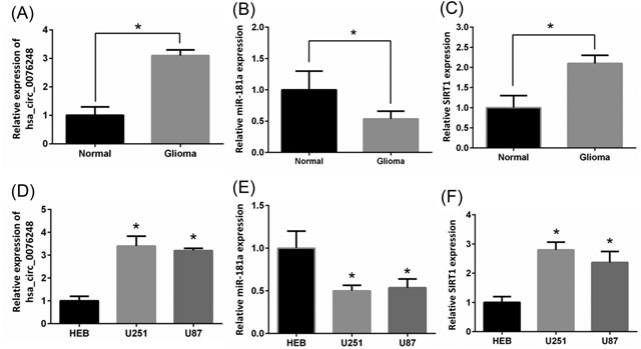
The expression of hsa_circ_0076248, miR‐181a, and SIRT1. A, qRT‐PCR detected the expression of hsa_circ_0076248 in human glioma tissues and normal brain tissues. B, qRT‐PCR detected the expression of miR‐181a in human glioma tissues and normal brain tissues. C, qRT‐PCR detected the expression of SIRT1 in human glioma tissues and normal brain tissues. D, qRT‐PCR detected the expression of hsa_circ_0076248 in human glioma cell lines U87, U251, and normal gliocyte HEB. E, qRT‐PCR detected the expression of miR‐181a in human glioma cell lines U87, U251, and normal gliocyte HEB. F, qRT‐PCR detected the expression of SIRT1 in human glioma cell lines U87, U251, and normal gliocyte HEB. *P < 0.05. qRT‐PCR, quantitative reverse‐transcription polymerase chain reaction; SIRT1, silent information regulator 1

To investigate the candidate role of miR‐181a in human glioma, we first detected whether the expression level of miR‐181a was downregulated in GBM tissues and cell lines. We found that the mRNA level of miR‐181a was markedly lower in GBM compared with the normal brain (Figure 1B). In addition, miR‐181a exhibited significantly lower expression in U87 and U251 glioma cell lines than HEB cell at mRNA level (Figure 1E). These data reflected that miR‐181a was decreased in human glioma. We checked the combination probability of hsa_circ_0076248, mir‐181a, and SIRT1 mRNA on the website of the National Center for Biotechnology Information (NCBI; Figure 6D) and then tested whether the expression level of hsa_circ_0076248 and SIRT1 was upregulated in GBM tissue and the cell lines. We found that the expression level of hsa_circ_0076248 and SIRT1 was markedly higher in GBM compared with the normal brain (Figure 1A and 1C). In addition, hsa_circ_0076248 and SIRT1 exhibited significantly higher expression in U87 and U251 glioma cell lines than HEB cell (Figure 1D and 1F). These data reflected that hsa_circ_0076248 and SIRT1 showed increased expression in human glioma.

### 3.2 MiR‐181a inhibited glioma cell proliferation and promoted apoptosis in vitro and circ_0076248 can reverse it

**Figure 2:**
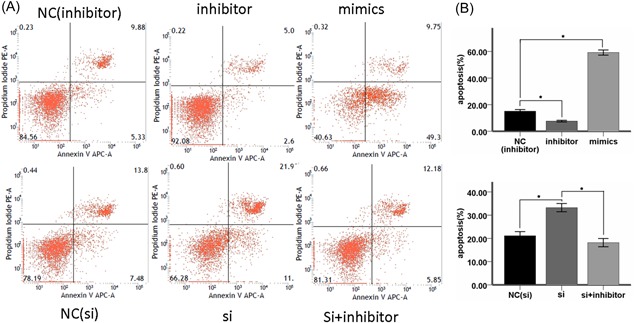
Cell apoptosis test. A, Annexin V‐APC‐A/PI apoptosis assay for U251 cells induced by 300 μM TMZ for 72 hours in miR‐181a‐inhibitor negative‐control group, miR‐181a‐inhibitor group, miR‐181a‐mimics group, si‐hsa_circ_0076248 negative‐control group, si‐hsa_circ_0076248 group and si‐hsa_circ_0076248 + miR‐181a‐inhibitor group individually. B, The statistical graph for U251 apoptosis assay induced by 300 μM TMZ showed miR‐181a‐inhibitor could remarkably depress the TMZ chemotherapy sensitivity; miR‐181a‐mimics and si‐hsa_circ_0076248 could remarkably promote the TMZ chemotherapy sensitivity; and si‐hsa_circ_0076248 + miR‐181a‐inhibitor could reverse the function of si‐hsa_circ_0076248 compared with negative‐control group; *P < 0.05. miR, microRNA; PI, propidium iodide; Si, small interfering; TMZ, temozolomide

**Figure 3:**
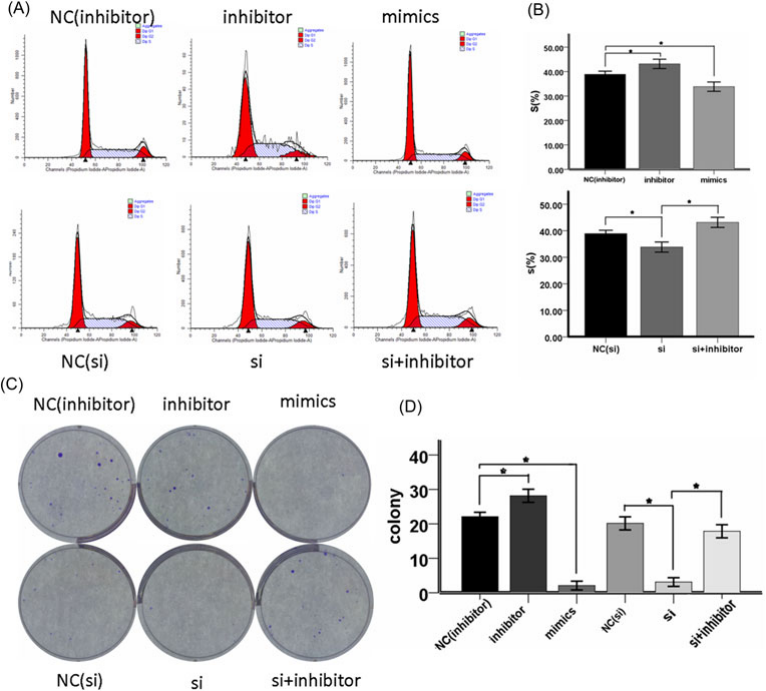
The cell cycle test and cell colony formation assay. A, Proportion of period of cell cycle for miR‐181a‐inhibitor negative‐control group, miR‐181a‐inhibitor group, miR‐181a‐mimics group, si‐hsa_circ_0076248 negative‐control group, si‐hsa_circ_0076248 group, and si‐hsa_circ_0076248 + miR‐181a‐inhibitor group individually. B, The statistical graph showed that the miR‐181a‐mimics and the si‐hsa_circ_0076248 blocked the cell cycle, while the miR‐181a‐inhibitor accelerated it, and si‐hsa_circ_0076248 + miR‐181a‐inhibitor reversed the function of si‐hsa_circ_0076248 compared with negative‐control group; *P < 0.05. C, Cell colony formation assay for miR‐181a‐inhibitor negative‐control group, miR‐181a‐inhibitor group, miR‐181amimics group, si‐hsa_circ_0076248 negative‐control group, si‐hsa_circ_0076248 group, and si‐hsa_circ_0076248 + miR‐181a‐inhibitor group, individually. D, The statistical graph which represents the average of triplicate experiments showed that the miR‐181a‐mimics and the si‐hsa_circ_0076248 depressed the proliferation, while the miR‐181a‐inhibitor‐promoted it, and si‐hsa_circ_0076248 + miR‐181a‐inhibitor reversed the function of si‐hsa_circ_0076248 compared with negative‐control group; *P < 0.05. MiR, microRNA

To investigate the biological function of miR‐181a in glioma tumorigenesis, Cell proliferation assay and cell colony formation assay were performed in U251 glioma cell lines. The result showed miR‐181a obviously decreased the proliferation (Figure 3A‐D) and promoted the apoptosis (Figure 2A and 2B) of U251 compared to the control cells. On the contrary, miR‐181a inhibitor^43^ (Genepharma) could promote U251 cell proliferation (Figure 3A and 3C) and inhibited cell apoptosis (Figure 2A). When we processed the cells with si‐hsa_circ_0076248, the situations of cell proliferation and cell colony formation, and apoptosis were strongly like what occurred after processing with miR‐181a, which attested the probable combination between hsa_circ_0076248 and miR‐181a based on their sequence (Figure 6D). Nude mice xenograft tumors were performed to verify the role of hsa_circ_0076248 on glioma proliferation in vivo. The result showed that downregulated hsa_circ_0076248 significantly suppressed the xenograft tumors' growth compared with the control group.

### 3.3 The relationship between hsa_circ_0076248, miR‐181a, and SIRT1

The sequence and the combination site of hsa_circ_0076248, miR‐181a, and mRNA of SIRT1 were identified on the website of NCBI (Figure 6D). The qPCR was performed to make sure the hsa_circ_0076248 works (Figure 6E), which showed that the expression of hsa_circ_0076248 was downregulated while the expression of mir‐181a was upregulated with no significant difference of SIRT1 mRNA.

### 3.4 The miR‐181a inhibited invasion and migration of glioma cell while the circ_0076248 reverse it

**Figure 4:**
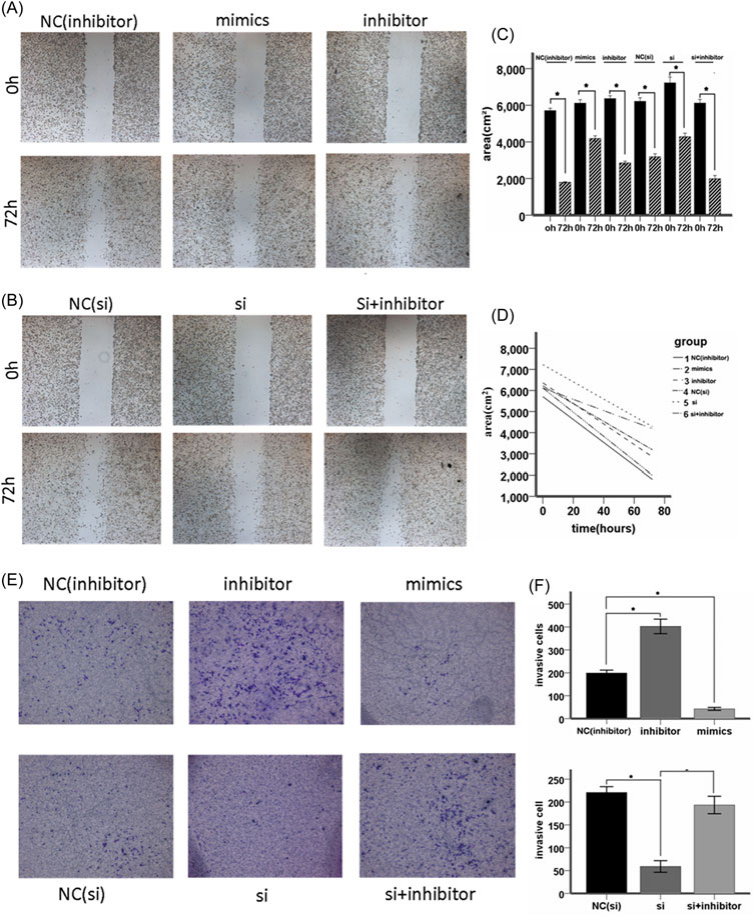
The scratch test and the transwell invasion test. A, The scratch test for miR‐181a‐inhibitor negative‐control group, miR‐181a‐inhibitor group, and miR‐181a‐mimics group individually in 72 hours. B, The scratch test for si‐hsa_circ_0076248 negative‐control group, si‐hsa_circ_0076248 group, and si‐hsa_circ_0076248 + miR‐181a‐inhibitor group, individually in 72 hours. C, D, The statistical graph showed that the miR‐181a‐mimics and the si‐hsa_circ_0076248 supressed the cell migration, while the miR‐181a‐inhibitor accelerated it, and si‐hsa_circ_0076248 + miR‐181a‐inhibitor reversed the function of si‐hsa_circ_0076248 compared with negative‐control group; *P < 0.05. E, The transwell test for miR‐181a‐inhibitor negative‐control group, miR‐181a‐inhibitor group, miR‐181a‐mimics group, si‐hsa_circ_0076248 negative‐control group, si‐hsa_circ_0076248 group, and si‐hsa_circ_0076248 + miR‐181a‐inhibitor group in 24 hours individually. F, The statistical graph showed that the miR‐181a‐mimics and the si‐hsa_circ_0076248 supressed the cell invasion, while the miR‐181a‐inhibitor accelerated it, and si‐hsa_circ_0076248 + miR‐181a‐inhibitor reversed the function of si‐hsa_circ_0076248 compared with negative‐control group; *P < 0.05. MiR, microRNA; si, small interfering

**Figure 5:**
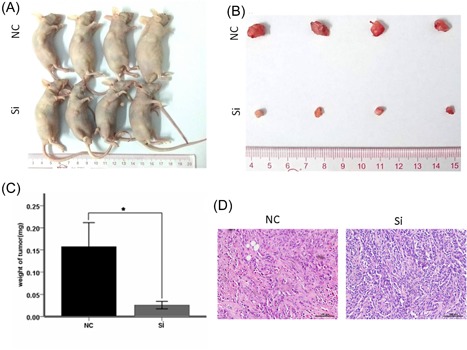
Subcutaneous tumor formation assay. A, B, Comparison of subcutaneous implants between control group and experimental group, it shows that the tumor of experimental group is smaller than the control group, to say, downregulate hsa_circ_0076248 inhibited glioma proliferation in subcutaneous tumor formation assay. C, The weight of tumor for NC group and si group; error bars depict mean ± SEM; data are from four independent experiments (four BALB/c nude) with independent *t* tests; D, HE dye for subcutaneous, which shows that the apoptosis tumor cell with disappearing of nucleus and filled with vacuole in the experimental group. ×20, object lens; ×10, observation lens. HE, hematoxylin and eosin; NC, negative control; SEM, standard error of the mean; Si, small interfering

To verify the probably combination between miR‐181a and hsa_circ_0076248, we test the transwell invasion test and scratch test (Figure 4A, 4B, and 4E). The result showed that the miR‐181a inhibited the migration and invasion of glioma cells and after we processed cells with si‐hsa_circ_0076248, which had been proved to downregulate the expression of hsa_circ_0076248 with qRT‐PCR, the result of transwell invasion test and scratch test were similar (Figure 4C, 4D, and 4F), assistant to the probable combination between miR‐181a and hsa_circ_0076248 (Figure 5).

### 3.5 SIRT1 is the potential target of miR‐181a in glioma

**Figure 6:**
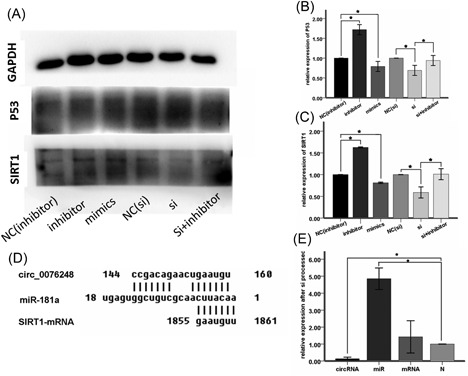
Western blot analysis and the binding site. A, The band of the GAPDH, P53, SIRT1 for miR‐181a‐inhibitor negative‐control group, miR‐181a‐inhibitor group, miR‐181a‐mimics group, si‐hsa_circ_0076248 negative‐control group, si‐hsa_circ_0076248 group and si‐hsa_circ_0076248 + miR‐181a‐inhibitor group individually. B, The statistical graph of relative integrated density of p53 showed that si‐hsa_circ_0076248 and the miR‐181amimics upregulated the expression of p53, while miR‐181a‐inhibitor downregulated it, and si‐hsa_circ_0076248 + miR‐181a‐inhibitor reversed the function of si‐hsa_circ_0076248 compared with negative‐control group. *P < 0.05. C, The statistical graph of relative integrated density of SIRT1 showed that si‐hsa_circ_0076248 and the miR‐181a‐mimics upregulated the expression of SIRT1, while miR‐181a‐inhibitor downregulated it, and si‐hsa_circ_0076248 + miR‐181a‐inhibitor reversed the function of si‐hsa_circ_0076248 compared with negative‐control group. *P < 0.05. D, the putative binding site between circ_0076248 and miR‐181a, miR‐181a, and SIRT1 mRNA. E, The relative expression of hsa_circ_0076248 was depressed after si processed while the relative expression of miR‐181a was upregulated and the mRNA no significance. GAPDH, glyceraldehyde 3‐phosphate dehydrogenase; miR, microRNA; mRNA, messenger RNA; SIRT1, silent information regulator 1

Bioinformatics analysis was performed using TargetScan software to identify the relationship among hsa_circ_0076248, miR‐181a and SIRT1; the result showed that miR‐181a could bind to the 3′‐UTR of SIRT1 mRNA, indicating that SIRT1 might be a potential target of miR‐181a. Results of Western blot analysis verified that overexpression of miR‐181a suppressed SIRT1 expression in U87 and U251 cells, while miR‐181a inhibitor could reverse the inhibition effect (Figure 6A‐C). qRT‐PCR results also confirmed that SIRT1 expression could be decreased by miR‐181a (Figure 6E). Real‐time RT‐PCR was performed to examine the mRNA level of Sirt1 in glioma and normal brain tissues. The expression of SIRT1 mRNA was significantly higher in glioma than that in normal brain tissues (Figure 1C). These data further supported that overexpression of miR‐181a would lead to the downregulation of SIRT1, which might further drive glioma growth and metastasis.

## 4 DISCUSSION

Glioma is one of the most common primary malignant tumors in the brain parenchyma.^44^, ^45^, ^46^ Malignant glioma has a poor prognosis despite the continuous progress in therapeutic technologies, including surgery, radiotherapy, photodynamic therapy, and chemotherapy.^47^ The high incidence and mortality of glioma prompt us to search for new therapeutic strategies.

Recent studies have disclosed various critical roles of circRNAs and miRNAs in cell growth and apoptosis.^15^ Each circRNA could sponge many miRNAs and each miRNA might have multiple target genes, and several miRNAs often regulated the same genes.^14^ The circRNAs and miRNAs performed important functions in various oncogenic characteristics, such as cell proliferation and migration.^48^ Human hsa_circ_0076248 also named hsa‐circ‐ZFAND3.7, one of the circular structures of ZFAND3 transcripts, had little research until nowadays, which could sponge many miRNAs including miR‐181a. Human miR‐181a was enriched in the brain^49^ and its aberrant expression has been associated with brain diseases.^43^ The expression of hsa‐miR‐181a was reduced in human gliomas and glioma cell lines, which was negatively correlated with tumor grade.^28^ Conversely, Chen et al^50^ indicated that miR‐181a sensitized human malignant glioma cells to radiation; however, the role of miR‐181a in human glioma chemoresistance, and more importantly, the downstream target and regulation mechanism that miR‐181a regulated human glioma tumorigenesis, still needed more exploration. SIRT1 affected a variety of biological functions, including DNA repair, energy metabolism, tumor suppression, and mitochondrial homeostasis.^30^, ^35^, ^37^, ^42^ These effects have been linked to cell behaviors, involving cell growth, differentiation, migration, and survival.^51^ To date, the expression and role of SIRT1 has been under investigation and its expression has been identified in various tumors, including breast cancer and liver cancer.^33^, ^34^ Our sample analysis identified that the expression of miR‐181a was lower in tumors than nontumor tissues. By contrast, hsa_circ_0076248 and SIRT1 showed much higher expression in tumor tissues than nontumor tissues. These findings were in agreement with previous findings. More importantly, downregulating hsa_circ_0076248 could remarkably promote the TMZ chemotherapy sensitivity, block the cell cycle, depress the proliferation in vitro and in vivo, suppress glioma cell invasion, and upregulate the expression of SIRT1, while miR‐181a inhibitor could reverse the effect. At the meantime, miR‐181a‐mimics shared the similar effect of downregulating hsa_circ_0076248. All the experiment verified the biological relationship among hsa_circ_0076248, miR‐181a, and SIRT1, which indicated that hsa_circ_0076248 could sponge miR‐181a which could suppress the expression of SIRT1.

The research of glioma treatment is facing with limited improvement as for the poor median survival time. The difficulty of glioma therapy accounts for the unidentified molecular mechanisms in diagnosis and treatment. Even though more and more biomarker and target of glioma emerged in recent years, such as IDH1, P53, MGMT, EGFR, nevertheless, none could reveal the general view of nosogenesis, as for its heterogenicity and complexity.^52^, ^53^, ^54^, ^55^ Taken together, our study illuminated the regulatory mechanism of hsa_circ_0076248 in glioma oncogenesis via the mediation of SIRT1 expression by sponging miR‐181a and suggested that hsa_circ_0076248 and miR‐181a as well as SIRT1 as potential therapeutic targets for future glioma therapy. In this study, the preliminary investigation on the function of hsa_circ_0076248 and the relationship among hsa_circ_0076248, miR‐181a, and SIRT1 will turn our eyes to noncoding RNA, especially circRNA, which may provide us with unexpected key on diagnosis and treatment of glioma.

## CONFLICTS OF INTEREST

The authors have declared that there are no conflicts of interest.
